# Interactions of Methylotrophs with Plants and Other Heterotrophic Bacteria

**DOI:** 10.3390/microorganisms3020137

**Published:** 2015-04-02

**Authors:** Hiroyuki Iguchi, Hiroya Yurimoto, Yasuyoshi Sakai

**Affiliations:** Division of Applied Life Sciences, Graduate School of Agriculture, Kyoto University, Kitashirakawa-Oiwake, Sakyo-ku, Kyoto 606-8502, Japan; E-Mails: iguchih@kais.kyoto-u.ac.jp (H.I.); yury@kais.kyoto-u.ac.jp (H.Y.)

**Keywords:** plant colonization, symbiosis

## Abstract

Methylotrophs, which can utilize methane and/or methanol as sole carbon and energy sources, are key players in the carbon cycle between methane and CO_2_, the two most important greenhouse gases. This review describes the relationships between methylotrophs and plants, and between methanotrophs (methane-utilizers, a subset of methylotrophs) and heterotrophic bacteria. Some plants emit methane and methanol from their leaves, and provide methylotrophs with habitats. Methanol-utilizing methylotrophs in the genus *Methylobacterium* are abundant in the phyllosphere and have the ability to promote the growth of some plants. Methanotrophs also inhabit the phyllosphere, and methanotrophs with high methane oxidation activities have been found on aquatic plants. Both plant and environmental factors are involved in shaping the methylotroph community on plants. Methanotrophic activity can be enhanced by heterotrophic bacteria that provide growth factors (e.g., cobalamin). Information regarding the biological interaction of methylotrophs with other organisms will facilitate a better understanding of the carbon cycle that is driven by methylotrophs.

## 1. Introduction

Methane and methanol, which are called C1 compounds, are ubiquitous in nature and important intermediates in the global carbon cycle. Methane is produced in anaerobic environments by methanogenic archaea, and is also generated abiotically by biomass burning, coal mining, and the oil industry. Methane is the second most important greenhouse gas after CO_2_, and its annual emission is estimated to be 580 Tg [1], an amount that has been increasing since the industrial era, and as such, the reduction of atmospheric methane has become a major social demand. It was discovered in 2006 that plant cells produce methane (12–370 ng per g dry weight h^−1^) [2], which is distinct from the microbially produced methane that is transported by hydrophytes from underground. Subsequent studies showed that various terrestrial plant species produce methane from pectin at significant rates corresponding to 2%–12% of the total global methane release [3,4]. Methanol is a volatile atmospheric carbon compound and its annual emission is estimated to be 150 Tg [5]. Methanol is principally generated from plant pectin and lignin during plant growth and decay (1.5–45.7 μg per g dry weight h^−1^) [6,7].

Methylotrophs are defined as microbes that can utilize C1 compounds as the sole source of carbon and energy. They include bacteria, yeasts, fungi, and archaea. Methanotrophs, which are the methane-utilizing methylotrophs, are limited to aerobic members of the γ*-Proteobacteria*, α*-Proteobacteria* and *Verrucomicrobia*, and anaerobic archaea and “*Candidatus* Methylomirabilis oxyfera” [8,9]. Almost all methanotrophs are obligate C1 utilizers, *i.e.*, they are only capable of growth on methane or methanol [10,11]. In the last decade, some methanotroph species in the genera *Methylocella*, *Methylocapsa* and *Methylocystis* were found to be facultative methanotrophs that can utilize organic acids (e.g., acetate) and ethanol [12,13,14]. On the other hand, the other group of methylotrophs, the methanol-utilizing methylotrophs, belong to diverse phyla, and most of them are facultative methylotrophs capable of growth on both single-carbon and multi-carbon compounds [15].

Most of the methane that is released into the atmosphere is oxidized by hydroxyl radicals in the troposphere [16]. Methanotrophs are the only biological sink for methane: they oxidize methane that is released from terrestrial and aquatic environments before it reaches the atmosphere, as well as atmospheric methane [17]. Methanotrophs convert methane into organic compounds, which are further utilized by other organisms. Stable isotope probe (SIP) experiments showed that the methane-derived carbon compounds were found not only in methanol-utilizing methylotrophs and other heterotrophic bacteria, but also in fungi, protists, mussels, and plants [18,19,20,21,22,23]. Therefore, methanotrophs play a critical role in incorporating the carbon atom of methane into the global carbon cycle.

Since some plants emit methane and methanol, the phyllosphere, defined as the aerial part of plants, has been recognized as a habitat for methylotrophs. Phyllospheric methanol-utilizing methylotrophs were discovered in the 1980s [24], and since then, our understanding of their ecology and physiology has increased. On the other hand, studies on phyllospheric methanotrophs progressed after a report in 2006 on methane emission from plants [2].

Here we review recent research advances demonstrating positive relationships between methylotrophs and plants, and between methanotrophs and heterotrophic bacteria.

## 2. Methanol-Utilizing Methylotrophs on Plants

### 2.1. Positive Effects of Methylobacterium Species on Plant Growth

The phyllosphere is a well-known habitat of methanol-utilizing methylotrophs, and leaf surfaces are colonized by a large population of these bacteria, which include the genera *Methylobacterium*, *Methylophilus*, *Methylibium* and *Hyphomicrobium* [25,26,27]. *Methylobacterium* species, which represent the main genus among the leaf microbial community, are known to have mutualistic relationships with some plants [28,29,30]. In summary, they are known to promote plant growth through plant hormone production and nutrient uptake support. In addition, they may affect plant health by suppressing pathogen growth and inducing systemic resistance [31]. These abilities of *Methylobacterium* species can be used in agricultural plant cultivation [32,33,34,35]. Further information regarding the positive effects of *Methylobacterium* species on plant growth can be found in several excellent articles [36,37].

### 2.2. Methylobacterium Community in the Phyllosphere

Many factors are involved in shaping the microbial community in the phyllosphere. It is known that phyllosphere microorganisms come from seeds, neighboring plants, soil, air, and aerosols [28,37]. In terms of bacterial traits, growth, nutrient availability, stress resistance, attachment, and motility contribute to colonization and survival on plants [37]. Among the limited carbon sources present on plant leaves [38,39], methanol is assumed to be abundant [40], which provides an advantage for colonization of methanol-utilizing methylotrophs on plants [41]. Also, the microbial community composition on leaves is affected by the plant genotype, plant age, soil type, climate and geography, the major driving forces being the plant genotype and geography [28,42,43,44].

The *Methylobacterium* community from leaves of *Arabidopsis thaliana*, *Medicago truncatula*, and surrounding plant species at five sampling sites located at distances ranging from 19 to 190 km was analyzed by culture-independent metagenome sequencing [45]. The results showed that the site of cultivation and plant species had strong effects on *Methylobacterium* community composition in the phyllosphere. The *Methylobacterium* communities of *Arabidopsis* plants from a given site were more similar to those of other plant species from the same site than to communities of *Arabidospsis* plants from a different site. The *Methylobacterium* population represented a constant fraction of the phyllosphere bacterial population.

Plant species affect the population size of *Methylobacterium* in the phyllosphere [45,46]. Leaves of vegetable species that were cultivated proximately in a home garden (*ca.* 100 m^2^) were found to each harbor a different *Methylobacterium* population [46]. Green perilla and eggplant harbored a large population of 10^7^ CFU/g fresh weight, while tomato and okra leaves showed populations of 10^5^ CFU/g fresh weight. Green perilla leaves purchased at some supermarkets also had populations comparable to those on leaves from the garden. These findings indicate that the plant species affects the size of *Methylobacterium* populations on leaves.

Efficient plant colonization by *Methylobacterium* was shown to be closely linked to bacterial phylogeny, by competitive tests for colonization of *Arabidopsis* plants by various *Methylobacterium* strains [47]. The phylogenetic group consisting of the species *M. radiotolerans*, *M. mesophilicum*, and *M. fujisawaense* represent strong colonizers. Specific interactions at the species level, with the association of specific *Methylobacterium* species with specific plant species, were observed [48]. The seeds and leaves of red perilla (*Perilla frutescens crispa* (Thunb.) Makino) harbored a large population of *Methylobacterium*, dominated by *M. fujisawaense*-related strains, whereas green perilla (*Perilla frutescens viridis* (Makino) Makino) harbored some *Methylobacterium* species (*M. radiotolerans*, *M. fujisawaense* and *M. komagatae*). When red perilla was cultivated at four distantly located sites (four prefectures in Japan) using locally purchased soils, almost all *Methylobacterium* strains isolated from the harvested leaves and seeds had 16S rRNA gene sequences (1.5 kb) identical to those of *Methylobacterium* sp. OR01, which was isolated from the parental seeds. This specific association was replicated in a second year trial using seeds harvested in the first year. These results suggested the following life cycle: (1) *M. fujisawaense*-related cells attached to red perilla seeds competitively proliferate in soils as plants grow, followed by colonization of leaves, ultimately becoming major members of the leaf microbial community; (2) the *Methylobacterium* cells are closely attached to seeds and are inherited by the next generation of plants. In this case, plant species influences the dominant *Methylobacterium* species regardless of geographical and environmental factors.

## 3. Plant-Associated Methanotrophs

### 3.1. Characteristics of Methanotrophs Inhabiting the Phyllosphere

In contrast to methanol-utilizing methylotrophs, knowledge of methanotrophs in the phyllosphere is limited. The finding of methane emission from plants [2] provided the idea that in addition to methanol, methanotrophs can utilize methane on leaf surfaces. The phyllosphere represents a unique environment [49] in comparison with major methanotroph habitats such as soil, lakes, and wetlands.

The first question was whether methanotrophs inhabit the phyllosphere. Attempts were made to cultivate methanotrophs from natural plant samples with mineral medium and methane. Cultivable methanotrophs were present on leaves and flowers of woody and herbaceous plants at relative high frequency (12%, 41/336 sample) [50]. Doronina *et al.* cultivated methanotrophs from linden buds and spruce needles [51]. Studies of bacterial communities using a culture-independent metagenomic technique detected methanotrophs on leaves of soybeans, rice, and *Tamarix* [52,53,54]. The appearance frequencies of sequences assigned to methanotrophs estimated that methanotrophs are minor members of the population (below 1%) in the phyllosphere community. Unfortunately, many metagenomic studies have not described the minor members, and thus little information has been obtained about phyllospheric methanotrophs, despite increasing amounts of metagenome data from plant samples.

**Figure 1:**
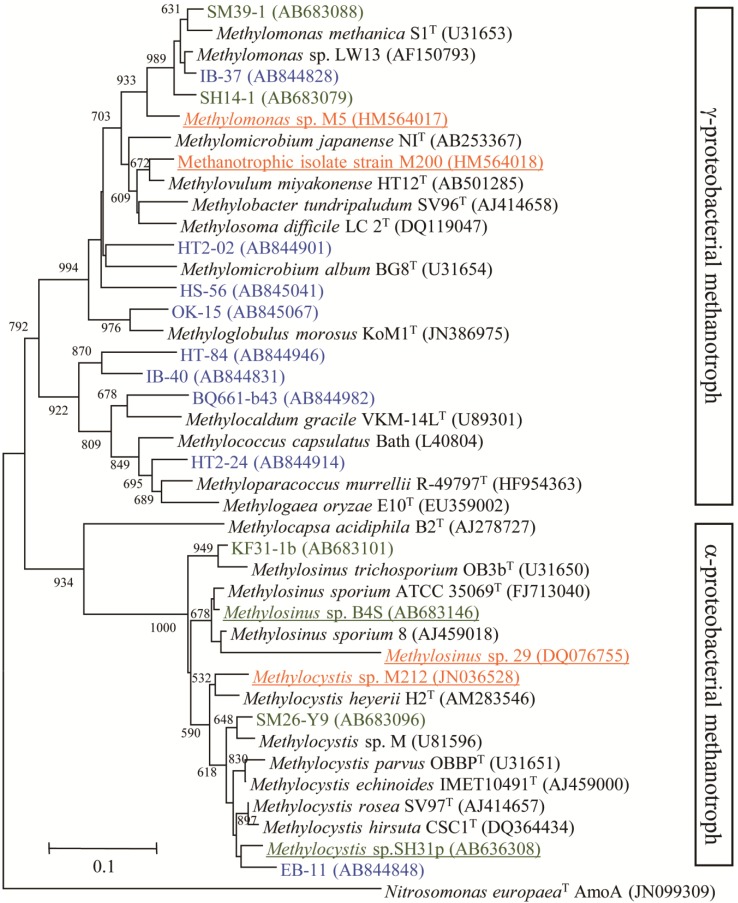
Phylogenetic tree of PmoA sequences from plant-derived methanotrophic isolates (bold letters) and methanotrophic clones retrieved from cultures. Methanotrophs that originated from plant samples are shown in colored letters: Plant tissues in the phyllosphere (green, [50]), macrophytic algae (blue, [56]) and *Sphagnum* mosses (orange, [57]). Bar, 0.1 substitutions per amino acid position.

Cultured methanotrophs from phyllosphere samples included γ- and α-*Proteobacteria* in the genera *Methylomonas*, *Methylobacter*, *Methylosinus*, and *Methylocystis* [50,51] (Figure 1). Up to three methanotroph species were present in each plant leaf sample. Metagenome sequencing revealed similar methanotrophs in the genera *Methylobacter*, *Methylococcus*, and *Methylosinus* [52,53,54]. These phyllospheric methanotrophs were neither affiliated with novel species nor belonged to phyllosphere-specific phylogenetic lineages. Instead, these genera have been found widespread in a variety of environments [8,55] and are assumed to be major players in the carbon cycle.

Growth of methanotrophs in the phyllosphere were analyzed by inoculating the leaf isolate *Methylosinus* sp. B4S on *Arabidopsis* leaves [58]. The inoculated methanotroph did not proliferate on leaves, but survived at least for 15 days, based on GFP expression and enumeration of methanotroph cells. This property can partially explain the high prevalence of methanotrophs with small population densities on leaves.

Methanotrophs use two types of methane monooxygenase (MMO) enzymes, particulate methane monooxygenase (pMMO) and soluble methane monooxygenase (sMMO), depending on copper availability in the environment [11,59]. Transcription analysis of MMO genes in *Methylosinus* sp. B4S showed higher expression of *mmoX* (encoding a subunit of sMMO) than that of *pmoA* (encoding a subunit of pMMO) on leaves [58]. This indicates that the plant leaf is an environment where the bioavailability of copper is low, allowing expression of sMMO genes.

PhyR, a regulator of the general stress response, was first identified as an abundantly produced protein in *Methylobacterium extorquens* AM1 and was shown to be necessary for plant colonization [60]. PhyR induces resistance to stresses such as heat, UV, reactive oxygen species, osmotic pressure, and drought [61,62], which are experienced in the phyllosphere. The *phyR* gene is distributed in α-*Proteobacteria* [63]. In the methanotroph *Methylosinus* sp. B4S, its function was also verified to regulate UV and heat resistances [58].

### 3.2. High Methane Oxidation by Methanotrophs on Aquatic Plants

Aqueous environments such as wetlands, paddy fields and lakes represent the largest source of methane (40%–50%) [1], in which much of the methane produced by methanogens is oxidized by methanotrophs before it can be released into the atmosphere. It is unclear whether macrophytes generate methane or methanol. Although methanotrophs have access to high concentrations of methane in these environments, allowing a large population, O_2_ availability is a limiting factor for methane oxidation. Since methane and O_2_ concentrations are oppositely correlated relative to water depth, methane oxidation activity is high at the oxic/anoxic interface [64].

Methanotrophs are known to associate with some aquatic plants in sediments and the water phase, and attention has been paid to the methane oxidation activity in the rhizosphere due to O_2_ release from roots [65,66]. It was recently revealed that shoots (including leaves) of aquatic plants are colonized by methanotrophs with high methane oxidation potentials. Among the tested shoot organs, fully-submerged macrophytic algae collected from a freshwater lake (*i.e.*, *Egeria densa*, *Cabomba caroliniana*, and *Chara braunii*) had much higher oxidation rates (3.7–37 μmol CH_4_ g^−1^·h^−1^) than emergent parts of terrestrial and aquatic plants [56]. This value is higher than that of rice roots (0.2–0.4 μmol CH_4_ g^−1^·h^−1^) [67]. Other studies reported methane oxidation of submerged macrophytic algae [68,69,70], and showed that shoots oxidize more methane than roots (*i.e.*, *Myriophyllum exalbescens*) [71].

The high methane oxidation potential of shoots is also found in the methanotroph-moss symbiotic system. Methane oxidation associated with *Sphagnum* mosses in peat bogs occurred at a significant rate (0.3–1.2 μmol CH_4_ g^−1^·h^−1^), and CO_2_ generated via methane oxidation provided 10%–30% of the carbon source for mosses [23]. The methanotrophs also provide accessible nitrogen (NH_4_^+^) for *Sphagnum* mosses by carrying out N_2_ fixation [72]. The vegetated environment of the mosses is an important factor affecting methane oxidation capacity [23,73,74]; rates were much higher in submerged moss than in non-submerged moss, although the methanotrophic communities were similar. It has been speculated that this is because of lower methane availability and higher stress levels in the aerial phase than the water phase.

In both the macrophytic algae and moss relationships, such high methane oxidation was attributed to a large population of associated methanotrophs [56,74,75]. No methane oxidation, indicating the absence of a significant methanotroph population, was observed in water from the vegetated environments. The associated methanotrophic community was different between the two plant ecosystems (Figure 1). Submerged macrophytic algae in the lake were dominated by γ-proteobacterial methanotrophs belonging to *Methylosarcina* and *Methylocaldum* [56], while *Sphagnum* mosses in peat bogs were dominantly associated with α-proteobacterial methanotrophs belonging to *Methylocystis* [57,73]. The methanotroph members were shown to move between mosses through water [75], indicating that mosses are a reservoir of methanotrophs. Together, vegetated environments for these aquatic plants can be a source of plant-associated methanotrophs, since γ- and α-proteobacterial methanotrophs are abundant in freshwater lake sediments and peat soils, respectively [22,76,77,78].

In summary, macrophytic algae and mosses in the aqueous phase are important habitats for methanotrophs. Plant-associated methanotrophs represent a significant population, and thus are expected to actively oxidize methane in natural environments. Such a large plant-associated population may be due in part to the availability of O_2_ provided by plants as well as the environment, which allows access to nutrients and protection from environmental stress.

## 4. Stimulation of Methanotrophic Activity by Heterotrophic Bacteria

Syntrophic relationships between methanotrophs and heterotrophic bacteria and between methanotrophs and methanol-utilizing bacteria have been long known. When environmental samples were incubated using methane as a sole carbon source in liquid culture, methanotrophs grew together with methanol-utilizing bacteria and other heterotrophic bacteria [55,79,80]. This relationship is represented as a methane-driven food web, in which methanotrophs provide other microbes with carbon compounds such as methanol, proteins (amino acids), polysaccharides, and nucleic acids [81,82]. However, information about the benefits that methanotrophs obtain from other bacteria is limited. One example is the removal of toxic methanol in batch cultures. Methanotrophs that are sensitive to methanol were rescued by removal of methanol from the culture by a methanol-utilizing *Hyphomicrobium* sp. [81]. Similarly, we observed that methane oxidation by the methanotroph *Methylocystis* sp. SS2C was initiated only after methanol in the culture was consumed by *Methylobacterium* sp. [83]. Explanations for the inhibition of methanotrophic growth by methanol include the low tolerance of methanotrophs to formaldehyde generated by the oxidation of methanol [80], and the competitive inhibition of MMO between methane and methanol [84].

Stimulation of methanotrophic growth by bacteria was investigated using a co-culture system with one methanotrophic strain and each of nine heterotrophic strains [79]. The bacterial strains that were used were from a methane-enrichment culture originating from a forest soil sample. Methane oxidation and growth of the methanotrophs were strongly stimulated by three rhizobia (*Rhizobium* sp., *Mesorhizobium* sp., and *Sinorhizobium* sp.). The growth stimulating factor produced by the partner strains was identified as cobalamin (vitamin B_12_). This cobalamin-dependent stimulation occurred for diverse γ-proteobacterial methanotrophic genera (*Methylovulum*, *Methyloparacoccus*, *Methylomonas*). These methanotroph strains did not produce cobalamin, and showed very weak or no growth in the absence of cobalamin, indicating that cobalamin is an essential growth factor. In some cases cobalamin auxotrophy may be a strain-specific effect because of the observed difference in the cobalamin-requirement for growth between *Methyloparacoccus murrellii* strains OS501 and R-49797 [85]. Accordingly, some γ-proteobacterial methanotrophs depend on cobalamin produced by bacteria and archaea in natural environments [86].

A search of the genome of *Mvul. miyakonense* HT12 (Accession: NZ_AQZU00000000) identified three candidate genes encoding cobalamin-dependent enzymes [86,87], ethanolamine ammonia-lyase, methionine synthase, and ribonucleotide reductase. The genes encoding cobalamin-independent enzymes for the latter two enzymes were also present in the genome. The physiological function of cobalamin in γ-proteobacterial methanotrophs needs further investigation.

Further co-culture experiments were carried out between nine methanotrophic strains and 25 heterotroph strains [88]. The tested methanotrophs could grow on methane without external cobalamin. Heterotrophic strains exerted positive or negative effects on the growth of co-culture depending on the methanotroph tested. No single heterotroph had a positive relationship with all of the tested methanotrophs. The strongest growth stimulation was observed in the co-culture of *Methylomonas* sp. M5 and *Cupriavidus taiwanensis* LMG 19424. Co-cultures consisting of γ-proteobacterial methanotrophs (*Methylomonas* sp. R-45363 and R-45383, and *Methylosarcina fibrate* DSM 13736) showed highly stimulated growth. Based on genomic analysis, this stimulation was correlated with the ability of the partner heterotroph to synthesize quinone, pyridoxine, and cobalamin.

The two studies described above [79,88] showed that growth of methanotrophs was supported by the specific heterotrophs. On the other hand, Ho *et al.* revealed that heterotroph diversity affected the methane oxidation rate [89]. The heterotroph diversity in the consortia (that is heterotroph richness) was generated by selecting up to 10 heterotrophic species in two phyla (*Proteobacteria* and *Firmicutes*). Increased numbers of heterotroph species in the consortia, regardless of the heterotroph combination, significantly stimulated methane oxidation by *Methylomonas methanica*. Methane oxidation was not affected by any single heterotrophs, and was decreased by addition of heterotroph spent medium. Therefore, heterotroph richness may provide versatility in metabolic capacity to relieve accumulated inhibitory compounds, thus enhancing methane oxidation. These findings imply that community-level interactions between methanotrophs and heterotrophs contribute to methane oxidation in natural ecological systems.

## 5. Conclusions and Perspective

**Figure 2:**
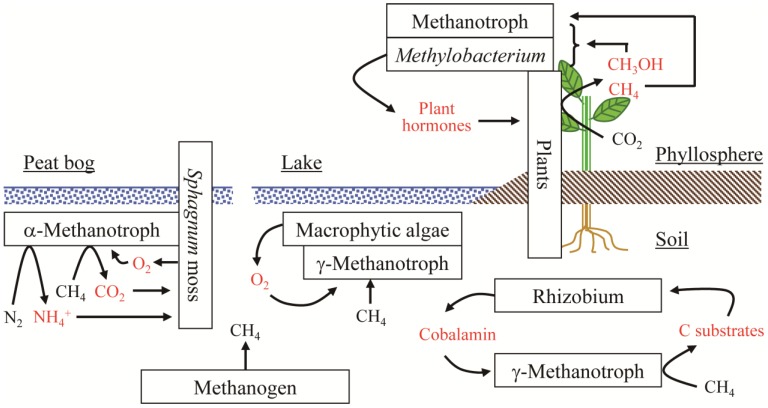
Positive relationships of methylotrophs. In the phyllosphere, C1 compounds generated by plants allow methanol-utilizing methylotrophs (e.g., *Methylobacterium* species) and methanotrophs to populate. In aquatic environments, methanotrophs belonging to γ- and α-*Proteobacteria* dominantly colonize on macrophytic algae and *Sphagnum* mosses, respectively, which supply O_2_ for methane oxidation by the methanotrophs. In several environments including soils, growth of some γ-proteobacterial methanotrophs require cobalamin supplied from heterotrophic bacteria such as rhizobial species.

Methylotrophs inhabit soil, water, and plants, and drive the carbon cycle through interaction with neighboring organisms such as plants and bacteria (Figure 2). Plants provide methylotrophs with habitats as well as compounds including growth substrates. In the phyllosphere, *Methylobacterium* species utilize methanol emitted by plants and affect plant growth. Methanotrophs, despite their small populations, also proliferate or survive in the phyllosphere. Recent studies revealed that the population and community composition of *Methylobacterium* are affected by plant genotype and vegetated environments. However, information available regarding the traits of plants and bacteria that are involved in shaping the methylotroph community on plants is limited. The strategies and mechanisms used by methylotrophs to select and to adapt to host plants remain to be determined.

Methanotrophs have interactions with plants under water through the exchange of excreted compounds. A much larger population of methanotrophs is assembled on macrophytes than in the water column. Macrophytes provide O_2_ for methane oxidation, while methanotrophs provide macrophytes with CO_2_ and NH_4_^+^. This mutualism helps to increase the biomass of both methanotrophs and macrophytes, thus stimulating methane oxidation in this ecosystem.

Trophic interactions between methanotrophs and heterotrophs have been observed in terrestrial and aquatic environments. Heterotrophic bacteria function as stimulators of methane oxidation by methanotrophs, e.g., through cobalamin production. This has important implications, which suggest that not only the methanotrophs but also microbial community members besides methanotrophs are involved in methane oxidation activity in the environment. Further studies of methylotrophs and their surrounding organisms and environments could result in the discovery of new biological interactions.
